# Supplementary material for: Family and personal history of cancer in the All of Us research program for precision medicine
Source: PLoS One. 2023 Jul 17;18(7):e0288496. doi: 10.1371/journal.pone.0288496 (PMC10351738; doi:10.1371/journal.pone.0288496)
Supplement: S1 Table — (DOCX) [file pone.0288496.s001.docx]

**S1 Table. *All of Us* Family History of Cancer by Demographic Categories Rates, Counts, and Ranking**.

|  |  | **Cancer Type Rates (n; rank)** | | | | | |  |
| --- | --- | --- | --- | --- | --- | --- | --- | --- |
| **Category** | **Subcategory** | **Breast** | **Colorectal** | **Lung** | **Ovarian** | **Prostate** | **Any 5** | |
| **Sex-at-birth** | Male | 13.8 (3975; 1) | 7.2 (2264; 4) | 8.7 (2567; 3) | 2.4 (739; 5) | 8.8 (2970; 2) | 28.9 (8589) | |
|  | Female | 13.4 (8186; 1) | 7.6 (4281; 4) | 8.6 (5160; 3) | 2.5 (1430; 5) | 10 (5190; 2) | 28.4 (16783) | |
| **Race &  Ethnicity** | Asian | 7.1 (207; 1) | 4.7 (135; 2) | 4.4 (127; 3) | 1 (30; 5) | 3.8 (111; 4) | 15.2 (441) | |
|  | Black | 11.9 (799; 1) | 6.6 (441; 4) | 7.5 (500; 3) | 2.3 (151; 5) | 8.6 (573; 2) | 25 (1676) | |
|  | Hispanic | 9.1 (632; 1) | 4.4 (302; 3) | 4.1 (283; 4) | 2.2 (152; 5) | 5.2 (360; 2) | 18 (1242) | |
|  | White | 14.7 (10161; 1) | 7.9 (5466; 4) | 9.5 (6588; 3) | 2.5 (1755; 5) | 9.9 (6862; 2) | 30.7 (21227) | |
|  | Other | 12.2 (456; 1) | 6.8 (255; 4) | 7.8 (291; 3) | 2.6 (97; 5) | 8.2 (307; 2) | 26.2 (976) | |
| **Age Group** | 20-29 | 5.5 (445; 1) | 1.9 (156; 3) | 1.4 (110; 4) | 1.1 (86; 5) | 2.3 (185; 2) | 9.5 (767) | |
|  | 30-39 | 7.8 (972; 1) | 2.7 (340; 4) | 2.8 (346; 3) | 1.6 (194; 5) | 4.2 (516; 2) | 14.7 (1825) | |
|  | 40-49 | 11.3 (1271; 1) | 5 (567; 4) | 5.7 (638; 3) | 2.3 (257; 5) | 7.5 (840; 2) | 23.5 (2652) | |
|  | 50-59 | 14.8 (2329; 1) | 7.9 (1246; 4) | 10.2 (1603; 3) | 2.5 (392; 5) | 10.4 (1633; 2) | 32 (5048) | |
|  | 60-69 | 17.8 (3948; 1) | 9.9 (2200; 4) | 12.8 (2847; 2) | 3.1 (685; 5) | 12.6 (2786; 3) | 38 (8430) | |
|  | 70-79 | 17 (2806; 1) | 10.7 (1765; 4) | 11.6 (1918; 3) | 3 (490; 5) | 11.9 (1956; 2) | 35.6 (5866) | |
|  | 80+ | 15.7 (476; 1) | 10.6 (321; 3) | 10.7 (324; 2) | 2.7 (81; 5) | 9.8 (296; 4) | 31.9 (963) | |
| **Income** | 0 - 25K | 10.2 (457; 1) | 5.5 (247; 3) | 6.7 (297; 2) | 2.8 (125; 5) | 4.6 (206; 4) | 20.3 (908) | |
|  | 25K - 50K | 12.2 (720; 1) | 6.8 (401; 4) | 8.4 (497; 2) | 2.4 (142; 5) | 7.2 (428; 3) | 25.6 (1515) | |
|  | 50K - 75K | 13.6 (1766; 1) | 7.5 (972; 4) | 9.1 (1175; 3) | 2.4 (307; 5) | 9.4 (1213; 2) | 28.9 (3751) | |
|  | 75K - 100K | 14 (1557; 1) | 8.3 (917; 4) | 9 (997; 3) | 2.6 (293; 5) | 9.9 (1102; 2) | 30 (3329) | |
|  | 100K - 150K | 14.6 (2139; 1) | 7.9 (1152; 4) | 8.8 (1294; 3) | 2.2 (321; 5) | 10.5 (1531; 2) | 30.5 (4466) | |
|  | 150K - 200K | 15.5 (1082; 1) | 7.7 (541; 4) | 8.7 (608; 3) | 2.2 (150; 5) | 10.6 (741; 2) | 31.3 (2190) | |
|  | > 200K | 16.1 (1557; 1) | 7.3 (704; 4) | 8.5 (824; 3) | 2.3 (218; 5) | 12.3 (1186; 2) | 33 (3196) | |
| **Education** | E1 | 8 (143; 1) | 5.3 (94; 3) | 6.4 (114; 2) | 2.9 (51; 5) | 4.7 (84; 4) | 18.4 (329) | |
|  | E2 | 12.3 (922; 1) | 7.5 (562; 3) | 9.9 (741; 2) | 2.9 (215; 5) | 6.1 (455; 4) | 25.7 (1923) | |
|  | E3 | 13.5 (2740; 1) | 7.6 (1547; 4) | 10 (2039; 2) | 2.7 (552; 5) | 8 (1628; 3) | 28.5 (5782) | |
|  | E4 | 14.1 (8387; 1) | 7.3 (4350; 4) | 8.2 (4843; 3) | 2.3 (1349; 5) | 10.1 (6001; 2) | 29.3 (17383) | |

E1 = Education Less than a high school degree or equivalent

E2 = Education Highest Grade: Twelve Or GED

E3 = Education Highest Grade: College One to Three

E4 = Education College graduate or advanced degree
