# Supplementary material for: Family and personal history of cancer in the All of Us research program for precision medicine
Source: PLoS One. 2023 Jul 17;18(7):e0288496. doi: 10.1371/journal.pone.0288496 (PMC10351738; doi:10.1371/journal.pone.0288496)
Supplement: S2 Table — (DOCX) [file pone.0288496.s002.docx]

**S2 Table. NHIS Family History of Cancer by Demographic Categories Rates, Counts, and Ranking.**

|  |  | **Cancer Type Rates (n; rank)** | | | | | |
| --- | --- | --- | --- | --- | --- | --- | --- |
| **Category** | **Subcategory** | **Breast** | **Colorectal** | **Lung** | **Ovarian** | **Prostate** | **Any 5** |
| **Sex-at-birth** | Male | 12.3 (1263; 1) | 7.3 (743; 4) | 9.5 (970; 2) | 2.4 (245; 5) | 7.6 (778; 3) | 32.8 (3364) |
|  | Female | 12.6 (1767; 1) | 7.6 (1074; 3) | 9.9 (1391; 2) | 2.7 (379; 5) | 7.1 (1004; 4) | 32.8 (4603) |
| **Race &**  **Ethnicity** | Asian | 8.5 (97; 1) | 4.8 (55; 3) | 6.6 (75; 2) | 1.2 (14; 5) | 4 (46; 4) | 21.6 (246) |
|  | Black | 10.7 (328; 1) | 6.8 (207; 4) | 8.2 (249; 2) | 2.2 (67; 5) | 7.7 (234; 3) | 28.8 (881) |
|  | Hispanic | 7.1 (262; 1) | 3.3 (121; 4) | 3.7 (138; 3) | 2 (73; 5) | 4 (149; 2) | 17.6 (652) |
|  | White | 14.4 (2232; 1) | 8.8 (1372; 3) | 11.8 (1835; 2) | 2.8 (440; 5) | 8.4 (1310; 4) | 38.2 (5931) |
|  | Other | 12 (89; 1) | 7.7 (57; 3) | 8.1 (60; 2) | 3.5 (26; 5) | 5.1 (38; 4) | 29.7 (221) |
| **Age Group** | 20-29 | 7.5 (147; 1) | 2.1 (40; 5) | 2.5 (48; 3) | 2.4 (46; 4) | 2.9 (57; 2) | 16.5 (321) |
|  | 30-39 | 6.1 (240; 1) | 2.8 (109; 4) | 3.4 (132; 2) | 1.6 (62; 5) | 3.3 (127; 3) | 15.7 (614) |
|  | 40-49 | 9 (370; 1) | 5.3 (217; 4) | 7 (286; 2) | 1.9 (76; 5) | 5.8 (239; 3) | 25.8 (1056) |
|  | 50-59 | 14.4 (682; 1) | 8.2 (389; 4) | 12.4 (588; 2) | 3 (141; 5) | 8.8 (417; 3) | 38.5 (1825) |
|  | 60-69 | 16.4 (752; 1) | 10.5 (482; 4) | 14.5 (662; 2) | 3.2 (145; 5) | 10.6 (484; 3) | 44.5 (2040) |
|  | 70-79 | 16.5 (503; 1) | 12.1 (368; 3) | 13.9 (424; 2) | 2.8 (86; 5) | 9.5 (291; 4) | 42.9 (1310) |
|  | 80+ | 16.9 (318; 1) | 11.1 (209; 3) | 11.7 (219; 2) | 3.4 (64; 5) | 8.7 (163; 4) | 41.1 (773) |
| **Income** | 0 - 25K | 10.4 (442; 1) | 5.3 (224; 4) | 7.9 (337; 2) | 2.2 (93; 5) | 5.6 (238; 3) | 26.5 (1125) |
|  | 25K - 45K | 10.2 (327; 1) | 5.9 (187; 4) | 7 (225; 2) | 2.1 (67; 5) | 6.1 (194; 3) | 27.1 (864) |
|  | 45K - 75K | 12.2 (345; 1) | 6.5 (185; 4) | 8.2 (233; 2) | 2.7 (75; 5) | 7.7 (218; 3) | 31.8 (899) |
|  | >75K | 14 (301; 1) | 7.5 (161; 4) | 9.1 (195; 3) | 2.7 (58; 5) | 9.2 (198; 2) | 35.5 (765) |
| **Education** | E1 | 9.4 (339; 1) | 5.7 (207; 3) | 9.3 (336; 2) | 2.7 (99; 5) | 4.8 (175; 4) | 26.3 (952) |
|  | E2 | 12.2 (758; 1) | 8.1 (502; 3) | 11 (681; 2) | 2.6 (160; 5) | 6.3 (390; 4) | 32.7 (2028) |
|  | E3 | 12.2 (547; 1) | 7.6 (340; 3) | 10.1 (453; 2) | 2.7 (119; 5) | 6.8 (305; 4) | 32.7 (1460) |
|  | E4 | 13.9 (1362; 1) | 7.7 (758; 4) | 9 (879; 3) | 2.4 (239; 5) | 9.2 (906; 2) | 35.4 (3474) |

E1 = Education Less than a high school degree or equivalent

E2 = Education Highest Grade: Twelve Or GED

E3 = Education Highest Grade: College One to Three

E4 = Education College graduate or advanced degree
