# Supplementary material for: Family and personal history of cancer in the All of Us research program for precision medicine
Source: PLoS One. 2023 Jul 17;18(7):e0288496. doi: 10.1371/journal.pone.0288496 (PMC10351738; doi:10.1371/journal.pone.0288496)
Supplement: S3 Table — (DOCX) [file pone.0288496.s003.docx]

**S3 Table: *All of Us* Personal History of Cancer by Demographic Categories Rates, Counts, and Ranking.**

|  |  | **Cancer Type Rates (n; rank)** | | | | | | |
| --- | --- | --- | --- | --- | --- | --- | --- | --- |
| **Category** | **Subcategory** | **Breast** | **Colorectal** | **Lung** | **Ovarian** | **Prostate** | **Any 5** |  |
| **Sex-at-birth** | Male | 1 (24; 4) | 12.3 (311; 2) | 6.4 (161; 3) | 0 (0; ) | 82.6 (2084; 1) | 34.6 |  |
|  | Female | 82.2 (3921; 1) | 8 (383; 2) | 5.7 (271; 4) | 6.9 (328; 3) | 0 (0; ) | 65.4 |  |
| **Race &  Ethnicity** | Asian | 64.4 (74; 1) | 7 (<20; 4) | 9.6 (<20; 3) | 6.1 (<20; 5) | 17.4 (20; 2) | 1.6 |  |
|  | Black | 53.9 (206; 1) | 10 (38; 3) | 5.8 (22; 5) | 6 (23; 4) | 27.2 (104; 2) | 5.3 |  |
|  | Hispanic | 63.5 (165; 1) | 13.9 (36; 3) | 5.4 (<20; 5) | 6.2 (<20; 4) | 14.6 (35; 2) | 3.6 |  |
|  | White | 53.4 (3377; 1) | 9.3 (589; 3) | 5.9 (372; 4) | 4.4 (276; 5) | 29.6 (1870; 2) | 87.3 |  |
|  | Other | 50 (<20; 1) | 7.1 (<20; 4) | 14.3 (<20; 3) | 3.6 (<20; 5) | 28.6 (<20; 2) | 0.4 |  |
| **Age Group** | 20-29 | 40 (<20; 1) | 26.7 (<20; 2) | 6.7 (<20; 4) | 26.7 (<20; 2) | 0 (0; 5) | 0.2 |  |
|  | 30-39 | 67 (73; 1) | 13.8 (<20; 3) | 4.6 (<20; 4) | 14.7 (1<20; 2) | 0 (0; ) | 1.5 |  |
|  | 40-49 | 76.2 (295; 1) | 10.1 (39; 3) | 3.6 (<20; 4) | 10.3 (40; 2) | 1.3 (<20; 5) | 5.3 |  |
|  | 50-59 | 69.6 (727; 1) | 10.54 (110; 2) | 4.6 (48; 5) | 7.5 (78; 4) | 10.2 (106; 3) | 14.2 |  |
|  | 60-69 | 56.8 (1340; 1) | 8.7 (206; 3) | 6.1 (143; 4) | 4.2 (100; 5) | 26 (611; 2) | 32 |  |
|  | 70-79 | 46.7 (1305; 1) | 9.2 (258; 3) | 6.3 (177; 4) | 2.8 (78; 5) | 38.2 (1062; 2) | 37.9 |  |
|  | 80+ | 36.2 (240; 2) | 10.1 (67; 3) | 8 (53; 4) | 2.7 (<20; 5) | 48 (318; 1) | 9 |  |
| **Income** | 0 - 25K | 53.5 (77; 1) | 17.4 (25; 2) | 9 (<20; 5) | 9.7 (<20; 4) | 12.5 (<20; 3) | 2.6 |  |
|  | 25K - 50K | 59.5 (657; 1) | 10.2 (111; 3) | 9 (66; 4) | 5.5 (62; 5) | 21.5 (410; 2) | 7.9 |  |
|  | 50K - 75K | 56 (321; 1) | 9.2 (47; 3) | 5.9 (30; 4) | 3.3 (21; 5) | 27.9 (200; 2) | 19.9 |  |
|  | 75K - 100K | 52.6 (257; 1) | 8.8 (44; 3) | 4.6 (39; 4) | 4 (24; 5) | 31.7 (93; 2) | 18.1 |  |
|  | 100K - 150K | 51.5 (608; 1) | 8.7 (100; 3) | 5.2 (64; 4) | 4.9 (36; 5) | 32.1 (303; 2) | 23.5 |  |
|  | 150K - 200K | 52.7 (518; 1) | 7.7 (87; 3) | 4.9 (45; 4) | 3.5 (39; 5) | 32.8 (312; 2) | 11.2 |  |
|  | > 200K | 49.8 (453; 1) | 6.6 (60; 3) | 4.3 (39; 4) | 2.9 (26; 5) | 37.7 (343; 2) | 16.7 |  |
| **Education** | E1 | 51.4 (38; 1) | 21.6 (<20; 2) | 10.8 (<20; 4) | 14.9 (<20; 3) | 6.8 (<20; 5) | 1 |  |
|  | E2 | 54.7 (290; 1) | 10.9 (58; 3) | 10.6 (56; 4) | 5.8 (31; 5) | 20 (106; 2) | 7.3 |  |
|  | E3 | 56.9 (910; 1) | 11.1 (178; 3) | 8 (128; 4) | 4.8 (77; 5) | 22.7 (363; 2) | 21.9 |  |
|  | E4 | 53.3 (2720; 1) | 8.6 (439; 3) | 4.8 (245; 4) | 4.1 (211; 5) | 31.5 (1610; 2) | 69.8 |  |

E1 = Education Less than a high school degree or equivalent

E2 = Education Highest Grade: Twelve Or GED

E3 = Education Highest Grade: College One to Three

E4 = Education College graduate or advanced degree
