# Supplementary material for: Family and personal history of cancer in the All of Us research program for precision medicine
Source: PLoS One. 2023 Jul 17;18(7):e0288496. doi: 10.1371/journal.pone.0288496 (PMC10351738; doi:10.1371/journal.pone.0288496)
Supplement: S4 Table — (DOCX) [file pone.0288496.s004.docx]

**S4 Table: NHIS Personal History of Cancer by Demographic Categories Rates, Counts, and Ranking.**

|  |  | **Cancer Type Rates (n; rank)** | | | | | |  |
| --- | --- | --- | --- | --- | --- | --- | --- | --- |
| **Category** | **Subcategory** | **Breast** | **Colorectal** | **Lung** | **Ovarian** | **Prostate** | **Any 5** | |
| **Sex-at-birth** | Male | 0.4 (2; 4) | 19.2 (92; 2) | 8.8 (42; 3) | 0 (0; 5) | 74.9 (359; 1) | 4.7 (479) | |
|  | Female | 74.4 (567; 1) | 12.2 (93; 2) | 6.7 (51; 4) | 9.5 (72; 3) | 0 (0; 5) | 5.4 (762) | |
| **Race &**  **Ethnicity** | Asian | 67.7 (21; 1) | 16.1 (5; 2) | 9.7 (3; 3) | 9.7 (3; 3) | 9.7 (3; 3) | 2.7 (31) | |
|  | Black | 38.3 (54; 1) | 12.8 (18; 3) | 10.6 (15; 4) | 7.1 (10; 5) | 32.6 (46; 2) | 4.6 (141) | |
|  | Hispanic | 40.7 (35; 1) | 8.1 (7; 4) | 5.8 (5; 5) | 10.5 (9; 3) | 36.1 (31; 2) | 2.3 (86) | |
|  | White | 46.8 (445; 1) | 15.9 (151; 3) | 7.3 (69; 4) | 5.1 (48; 5) | 28.2 (268; 2) | 6.1 (951) | |
|  | Other | 43.3 (13; 1) | 10 (3; 3) | 3.3 (1; 5) | 6.7 (2; 4) | 36.7 (11; 2) | 4 (30) | |
| **Age Group** | 20-29 | 50 (1; 1) | 0 (0; ) | 0 (0; ) | 50 (1; 1) | 0 (0; ) | 0.1 (2) | |
|  | 30-39 | 56.3 (9; 1) | 12.5 (2; 3) | 0 (0; ) | 37.5 (6; 2) | 0 (0; ) | 0.4 (16) | |
|  | 40-49 | 56.5 (26; 1) | 17.4 (8; 3) | 4.4 (2; 4) | 19.6 (9; 2) | 2.2 (1; 5) | 1.1 (46) | |
|  | 50-59 | 55 (88; 1) | 14.4 (23; 2) | 10 (16; 4) | 8.8 (14; 5) | 13.1 (21; 3) | 3.4 (160) | |
|  | 60-69 | 44.3 (147; 1) | 14.2 (47; 3) | 8.1 (27; 4) | 5.1 (17; 5) | 30.4 (101; 2) | 7.3 (332) | |
|  | 70-79 | 43.9 (169; 1) | 12 (46; 3) | 8.3 (32; 4) | 4.2 (16; 5) | 34 (131; 2) | 12.6 (385) | |
|  | 80+ | 43 (129; 1) | 19.7 (59; 3) | 5.3 (16; 4) | 3 (9; 5) | 35 (105; 2) | 16 (300) | |
| **Income** | 0 - 25K | 54.7 (70; 1) | 8.6 (11; 3) | 3.9 (5; 5) | 8.6 (11; 3) | 25 (32; 2) | 3 (128) | |
|  | 25K - 45K | 45.2 (28; 1) | 11.3 (7; 3) | 4.8 (3; 5) | 9.7 (6; 4) | 29 (18; 2) | 1.9 (62) | |
|  | 45K - 75K | 53.1 (34; 1) | 14.1 (9; 3) | 4.7 (3; 4) | 4.7 (3; 4) | 25 (16; 2) | 2.3 (64) | |
|  | >75K | 52.5 (32; 1) | 8.2 (5; 3) | 4.9 (3; 4) | 3.3 (2; 5) | 32.8 (20; 2) | 2.8 (61) | |
| **Education** | E1 | 40 (78; 1) | 14.9 (29; 3) | 12.3 (24; 4) | 6.2 (12; 5) | 29.2 (57; 2) | 5.4 (195) | |
|  | E2 | 45 (143; 1) | 18.6 (59; 3) | 6.6 (21; 5) | 7.2 (23; 4) | 27 (86; 2) | 5.1 (318) | |
|  | E3 | 47.1 (113; 1) | 17.9 (43; 3) | 7.5 (18; 4) | 6.3 (15; 5) | 23.8 (57; 2) | 5.4 (240) | |
|  | E4 | 47.9 (231; 1) | 11 (53; 3) | 6 (29; 4) | 4.1 (20; 5) | 33 (159; 2) | 4.9 (482) | |

E1 = Education Less than a high school degree or equivalent

E2 = Education Highest Grade: Twelve Or GED

E3 = Education Highest Grade: College One to Three

E4 = Education College graduate or advanced degree
