# Supplementary material for: Family and personal history of cancer in the All of Us research program for precision medicine
Source: PLoS One. 2023 Jul 17;18(7):e0288496. doi: 10.1371/journal.pone.0288496 (PMC10351738; doi:10.1371/journal.pone.0288496)
Supplement: S5 Table — (DOCX) [file pone.0288496.s005.docx]

**S5 Table. Conditional Probability of personal and familial history of cancer given a personal history of cancer with regards to Sex at Birth or Race and Ethnicity.**

|  |  |  | **% (PH & FH) given PH** | | **% (PH & FH) given FH** | |
| --- | --- | --- | --- | --- | --- | --- |
| **Category** | **Subcategory** | **Cancer Type** | ***AoU*** | **NHIS** | ***AoU*** | **NHIS** |
| Sex-at-birth | Female | Breast | 26.5 | 23.6 | 12.3 | 1.6 |
|  |  | Colorectal | 17.5 | 18.3 | 1.3 | 0.4 |
|  |  | Lung | 8.6 | 21.6 | 0.4 | 0.2 |
|  |  | Ovarian | 9.9 | 9.7 | 2 | 0.5 |
|  |  | Prostate | 0* | 0** | 0 | 0** |
|  |  | Any 5 | 34.2* | 38.9 | 9.4 | 1.7 |
|  | Male | Breast | 33.3 | 50* | 0.2 | 0.03* |
|  |  | Colorectal | 13.5 | 15.2* | 1.6 | 0.6* |
|  |  | Lung | 13.6 | 16.7* | 0.7 | 0.3* |
|  |  | Ovarian | 0** | 0** | 0 | 0** |
|  |  | Prostate | 3.1 | 22.3 | 2.1 | 2.7 |
|  |  | Any 5 | 34.2 | 36.9 | 9.8 | 2 |
| Race &  Ethnicity | Asian | Breast | 15.5 | 28.6 | 5.3 | 6.2 |
|  |  | Colorectal | 16.7* | 20* | 0.7* | 1.8* |
|  |  | Lung | 11.1* | 0* | 0.8* | 0* |
|  |  | Ovarian | 0 | 0* | 0 | 0* |
|  |  | Prostate | 0* | 0* | 0* | 0* |
|  |  | Any 5 | 37.3 | 29.6 | 9.3 | 3.3 |
|  | Black | Breast | 27 | 16.7 | 6.6 | 2.7 |
|  |  | Colorectal | 11.8 | 5.6* | 0.9 | 0.5* |
|  |  | Lung | 0* | 6.7* | 0* | 0.4* |
|  |  | Ovarian | 19.1 | 0* | 2.7 | 0* |
|  |  | Prostate | 1 | 21.7 | 0.2 | 4.3 |
|  |  | Any 5 | 28.5 | 35.3 | 6.3 | 5.6 |
|  | Hispanic | Breast | 24.5 | 25.7 | 6 | 3.4 |
|  |  | Colorectal | 36.7 | 14.3* | 3.6 | 0.8* |
|  |  | Lung | 10* | 20* | 0.4* | 0.7* |
|  |  | Ovarian | 0* | 11.1* | 0* | 1.4* |
|  |  | Prostate | 3.1 | 16.1 | 0.3 | 3.4 |
|  |  | Any 5 | 34 | 34.1 | 6.6 | 4.5 |
|  | Non-Hispanic  White | Breast | 26.9 | 24.7 | 8.7 | 4.9 |
|  |  | Colorectal | 14.7 | 17.2 | 1.4 | 1.9 |
|  |  | Lung | 10.8 | 23.2 | 0.5 | 0.9 |
|  |  | Ovarian | 9.3 | 10.4 | 1.3 | 1.1 |
|  |  | Prostate | 3.2 | 23.5 | 0.9 | 4.8 |
|  |  | Any 5 | 34.4 | 39.3 | 10 | 6.1 |
|  | Other | Breast | 26.7* | 7.7* | 0.9* | 1.1* |
|  |  | Colorectal | 50* | 33.3* | 0.4* | 1.8* |
|  |  | Lung | 0* | 0* | 0* | 0* |
|  |  | Ovarian | 100* | 50* | 1.0* | 3.9* |
|  |  | Prostate | 0* | 18.2* | 0* | 5.3* |
|  |  | Any 5 | 53.6* | 33.3 | 1.5* | 4.5 |

* < 20 responders in PH category; ** 0 responders in PH category
