# Supplementary material for: Family and personal history of cancer in the All of Us research program for precision medicine
Source: PLoS One. 2023 Jul 17;18(7):e0288496. doi: 10.1371/journal.pone.0288496 (PMC10351738; doi:10.1371/journal.pone.0288496)
Supplement: Attachment — Submitted filename: Response to Reviewers.pdf [file pone.0288496.s007.pdf]

UNIVERSITY of CALIFORNIA, SAN DIEGO
SCHOOL OF MEDICINE

DIVISION OF BIOMEDICAL INFORMATICS
DEPARTMENT OF MEDICINE
9500 GILMAN DR. SCHOOL OF MEDICINE
SAN DIEGO, CALIFORNIA 92093-0728
Phone: (858) 822-4931

June 12, 2023

Re: Dr. Michal Rosen-Zvi
Academic Editor
PLOS ONE

Dear Dr. Rosen-Zvi,

Please find the responses below for the review of our submission entitled: *Family and Personal History of Cancer in the All of Us Research Program for Precision Medicine*. Additionally, for us to comply with an *All of Us* policy that requires removal of participant counts less than 20 and prevention of the ability to calculate participant counts less than 20 from published material, in Supplemental Table 3, count (n) values for the 'Any 5' cancers column have been removed. We thank the reviewers for their insightful comments and constructive suggestions, and we are grateful for their time and effort. We have revised the manuscript to address the provided comments.

Sincerely,

Lauryn Keeler Bruce, Paulina Paul, Katherine K. Kim, Jihoon Kim, Theresa HM Keegan, Robert A. Hiatt, and Lucila Ohno-Machado
Submission ID: PONE-D-23-04133

Editor Comments

When submitting your revision, we need you to address these additional requirements.

1. Please ensure that your manuscript meets PLOS ONE's style requirements, including those for file naming. The PLOS ONE style templates can be found at https://journals.plos.org/plosone/s/file?id=wjVg/PLOSOne_formatting_sample_main_body.pdf and https://journals.plos.org/plosone/s/file?id=ba62/PLOSOne_formatting_sample_title_authors_affiliations.pdf

Response: Thank you, we have updated the formatting to comply with the links provided.

2. Please provide additional details regarding participant consent. In the ethics statement in the Methods and online submission information, please ensure that you have specified (1) whether consent was informed and (2) what type you obtained (for instance, written or verbal, and if verbal, how it was documented and witnessed). If your study included minors, state whether you obtained consent from parents or guardians. If the need for consent was waived by the ethics committee, please include this information.

If you are reporting a retrospective study of medical records or archived samples, please ensure that you have discussed whether all data were fully anonymized before you accessed them and/or whether the IRB or ethics committee waived the requirement for informed consent. If patients provided informed written consent to have data from their medical records used in research, please include this information.

Response: All data were collected from participants that were enrolled through the All of Us program. The program has anonymized all collected data. Additionally, this work was overseen by the program's Science Committee and confirmed as meeting the criteria for non-human subjects research by the *All of Us* Institutional Review Board.

3. Thank you for stating in your Funding Statement:
"L.K.B, K.K., and L.O.M are supported by a grant through the NIH Directors office (OT2OD026552). T.H.M.K is supported by a grant through the National Cancer Institute (P30CA093373)."

Please provide an amended statement that declares *all* the funding or sources of support (whether external or internal to your organization) received during this study, as detailed online in our guide for authors at http://journals.plos.org/plosone/s/submit-now. Please also include the statement "There was no additional external funding received for this study." in your updated Funding Statement.

Please include your amended Funding Statement within your cover letter. We will change the online submission form on your behalf.

Response: Thank you for the additional guidelines on declaring funding sources. As there is no additional funding for this study, the language "There was no additional external funding received for this study" is now included in the funding statement.

4. Thank you for stating the following financial disclosure:
"L.K.B, K.K., and L.O.M are supported by a grant through the NIH Directors office (OT2OD026552). T.H.M.K is supported by a grant through the National Cancer Institute (P30CA093373)."

Please state what role the funders took in the study. If the funders had no role, please state: ""The funders had no role in study design, data collection and analysis, decision to publish, or preparation of the manuscript.""
If this statement is not correct you must amend it as needed.
Please include this amended Role of Funder statement in your cover letter; we will change the online submission form on your behalf.

Response: Thank you for highlighting the need for funders' roles to be outlined in this section. None of the funders had roles in this study, thus the language "The funders had no role in study design, data collection and analysis, decision to publish, or preparation of the manuscript" is now included in the funding statement.

5. Thank you for stating the following in your Competing Interests section:
"No authors have competing interests"

Please complete your Competing Interests on the online submission form to state any Competing Interests. If you have no competing interests, please state ""The authors have declared that no competing interests exist."", as detailed online in our guide for authors at http://journals.plos.org/plosone/s/submit-now
This information should be included in your cover letter; we will change the online submission form on your behalf.

Response: Thank you for providing the correct language for this statement. The competing interests statement has been updated accordingly.

6. One of the noted authors is a group or consortium All of Us Consortium.. In addition to naming the author group, please list the individual authors and affiliations within this group in the acknowledgments section of your manuscript. Please also indicate clearly a lead author for this group along with a contact email address.

Response: The All of Us Program investigators are now mentioned in the acknowledgment section with reference to the full author list in Supplementary Information file S6.

7. Your ethics statement should only appear in the Methods section of your manuscript. If your ethics statement is written in any section besides the Methods, please move it to the Methods section and delete it from any other section. Please ensure that your ethics statement is included in your manuscript, as the ethics statement entered into the online submission form will not be published alongside your manuscript.

Response: Thank you for clarifying which section the ethics statement should reside. The statement has been moved to the Methods section.

8. Please include captions for your Supporting Information files at the end of your manuscript, and update any in-text citations to match accordingly. Please see our Supporting Information guidelines for more information: http://journals.plos.org/plosone/s/supporting-information.

Response: Thank you for providing the formatting guidelines for the supplementary information. The captions have been included at the end of the manuscript, in-text citations have been reformatted, and the supplementary tables have been split into five documents and named appropriately (e.g., S1_Table.docx).

9. Please review your reference list to ensure that it is complete and correct. If you have cited papers that have been retracted, please include the rationale for doing so in the manuscript text, or remove these references and replace them with relevant current references. Any changes to the reference list should be mentioned in the rebuttal letter that accompanies your revised manuscript. If you need to cite a retracted article, indicate the article's retracted status in the References list and also include a citation and full reference for the retraction notice.

Response: None of the cited articles have been retracted to our knowledge. One citation (Seabold 2010) had not been correctly added to the reference list. It is now listed as reference #14.

Additional Editor Comments:

This paper is well written and contributes to the understanding of cancer prevalence rates in US and associations of family and personal histories with these rates. It is based on analysis of All of Us data collection and compared with participants from the 2015 National Health Interview Survey (NHIS). It is a comprehensive intriguing analysis. The paper can be further improved as suggested by both reviewers. See their comments.

[Note: HTML markup is below. Please do not edit.]

Reviewers' comments:

Reviewer's Responses to Questions

Comments to the Author

1. Is the manuscript technically sound, and do the data support the conclusions?

The manuscript must describe a technically sound piece of scientific research with data that supports the conclusions. Experiments must have been conducted rigorously, with appropriate controls, replication, and sample sizes. The conclusions must be drawn appropriately based on the data presented.

Reviewer #1: Yes

Reviewer #2: Yes

---

2. Has the statistical analysis been performed appropriately and rigorously?

Reviewer #1: Yes

Reviewer #2: Yes

---

3. Have the authors made all data underlying the findings in their manuscript fully available?

The PLOS Data policy requires authors to make all data underlying the findings described in their manuscript fully available without restriction, with rare exception (please refer to the Data Availability Statement in the manuscript PDF file). The data should be provided as part of the manuscript or its supporting information, or deposited to a public repository. For example, in addition to summary statistics, the data points behind means, medians and variance measures should be available. If there are restrictions on publicly sharing data—e.g. participant privacy or use of data from a third party—those must be specified.

Reviewer #1: Yes

Reviewer #2: Yes

---

4. Is the manuscript presented in an intelligible fashion and written in standard English?

PLOS ONE does not copyedit accepted manuscripts, so the language in submitted articles must be clear, correct, and unambiguous. Any typographical or grammatical errors should be corrected at revision, so please note any specific errors here.

Reviewer #1: Yes

Reviewer #2: Yes

---

5. Review Comments to the Author

Please use the space provided to explain your answers to the questions above. You may also include additional comments for the author, including concerns about dual publication, research ethics, or publication ethics. (Please upload your review as an attachment if it exceeds 20,000 characters)

6. PLOS authors have the option to publish the peer review history of their article (what does this mean?). If published, this will include your full peer review and any attached files.

If you choose “no”, your identity will remain anonymous but your review may still be made public.

Do you want your identity to be public for this peer review? For information about this choice, including consent withdrawal, please see our Privacy Policy.

Reviewer #1: No

Reviewer #2: **Yes:** Vesna Barros

Reviewer #1

The manuscript describes important findings for rates of certain types of cancers such as breast, colorectal, lung, prostate etc. from AoU cohort and compares it with NHIS cohort from self-reported family history (FH) and personal history (PH) survey questionnaires. Overall, the paper is well written, but is rather long and can be described more concisely in Results. It's not clear what the main findings are – "we found similar family cancer history (33%) rates, but higher personal cancer history, with rates of personal cancer of nearly double in AoU as compared to the NHIS (9.2% vs. 5.11%)". If it's the latter, it will help the reader if they could describe results more concretely and point to the relevant tables.

Response: Thank you for the comment, we have simplified this statement in the Abstract.

1. Page 11- 314,994 individuals who completed 'The Basics' survey this number is larger in Table 1 (383,808) if its AoU v4

Response: Thank you for highlighting this discrepancy. The All of Us database v4 does have 383,808 participants, and of those, 314,994 individuals completed the Basics survey. We clarified this in the "All of Us Study Cohort" Section.

2. Table 2, footnote lists denominator as 89,458 whereas the text lists as 89,453 from AoU with FH

Response: Thank you for catching this discrepancy, 89,453 was the total n used in the code, and the typo has been fixed in the Table 2 footnote.

3. Similarly, for AoU with FH + PH lists 89,142 vs. 89,152 in the text

Response: Thank you for reporting this discrepancy. The correct value was 82152 participants that completed the personal cancer and history of cancer survey, thus the value in the Table 2 footnote was correct. The text has been changed to reflect the correct value.

4. Table 6, Page 22 – need N besides percentages for specific cancer types – in other words need to know how many in each bin for calculating conditional probability. This is the table that shows double rates?

Response: Thank you for requesting clarification. Table 6 contains all n values used for calculating the conditional probability. For example, the probability of FH of Breast cancer given the participant completed the PH survey is equal to 11814/82152 = 14.4%. For the rate of breast cancer for a participant given that they also have a family history (PH|FH) is equal to 1197 / 11814 = 10.1%.

5. Interestingly, colorectal cancer conditional probability remains same (2.20%) between AoU and NHIS cohorts in Table 6, while Breast cancer is doubled for AoU (10.10% vs 5.40%). Why such a discrepancy? It may be also good to discuss, if it's an artifact of data collection/survey methods?

Response: Thank you for highlighting this interesting difference. We do not believe the differences can be attributed to data collection methods.

Reviewer #2:

This interesting manuscript compares rates of personal cancer history and family cancer history in the All of Us database and in NHIS. The paper is well written and technically describes surveys on large enough datasets (383,808 in All of Us, and 76,261 in NHIS), as well as in sub-cohorts of these datasets. The authors performed several summary statistics of the data, which can be seen in the tables in the main text and supplemental material. The code and datasets are open and available for any researcher who registers and completes the ethics training of All of Us. Specific points to be addressed:

1. Materials and methods, Page 10: "with the hypothesis that prevalence statistics and trends would be comparable despite the differences in population composition". If the study is hypothesis-driven, it would be ideal to state the hypothesis in the introduction. Materials and methods should state only well-described methods that can effectively answer the questions you are addressing.

Response: Thank you for this comment. We have removed the hypothesis statement from the Materials and methods section.

2. Race and ethnicity categories were collected in the All of Us Research Program. Is it possible to also include the source of the classifications used (eg, self-report or selection, investigator observed, database, electronic health record, survey)?

Response: Thank you for requesting this clarification. All race and ethnicity data were self-reported in the 'The Basics' survey. We have now stated this in the methods section.

3. Section "All of Us Study Cohort": For those who are not familiar with the AoU database, what exactly does "the Basics" survey include? Which information does it contain? Please describe in one sentence or two.

Response: Thank you for highlighting the need for describing "The Basics" survey. Additional information has been added to the "All of Us Study Cohort" section.

4. Page 12, section "All of Us Dataset Preparation", 1st sentence: word input was repeated twice.

Response: Thank you for identifying that typographical error, this has now been resolved.

5. Statistical Analysis, page 13: When stating the statistical methods used to analyze the data (two-sample proportion z-test), also state the p-value used for significance (was it .05? Then add "A p-value less than .05 was considered to indicate statistical significance."

Response: Thank you for requesting this clarification, the statement suggested has been added to the statistical analysis section as a p-value of 0.5 is considered as statically significant for this study.

6. When mentioning statistical softwares/packages you used, also state version and manufacturer's name. Eg: We used numpy (version X.X.X, https://numpy.org) and pandas (version X.X.X., https://pandas.pydata.org). Same for jupyter notebook, and other libraries you used. This allows others to reproduce the study.

Response: Thank you for the suggestion, all versions and websites have now been added for the packages listed in the statistical analysis section.

7.Table 1: Spell out the abbreviation "FH" in the table legend. The abbreviations E1, E2, E3 were defined but they do not appear in the table.

Response: Thank you for the suggestion. 'FH' is no longer abbreviated and the E1-E3 definitions have been removed.

8. Maybe there is a typo in the first paragraph of Results: "the same level of diversity in seen". Did you mean "as seen"?

Response: Thank you for identifying this typo, we have replaced 'in' with 'as'

9. Provide statistical significance (p-values) when comparing variables (race/ethnicity, age, biological sex) between cohorts. This is important to verify that your statistical analyses are performed to a high technical standard and are described in sufficient detail.

Response: We are not able to compare cancer rates directly between the two cohorts as these populations are significantly different by a chi-squared test ($p << 0.01$) and it is known that the All of Us cohort was not designed to be representative of the population. We instead compare conditional probabilities of personal history given family history (Table 5, S5).

10. Under Results: "The demographic composition for the NHIS survey population and the FH subset are comparable for education and income while race and ethnicity are slightly skewed White, Female, and older." Please rephrase

this sentence. Perhaps " […] while race/ethnicity and biological sex are slightly skewed towards White and female, respectively"

Response: Thank you for the comment, the sentence has been rephrased as suggested.

11. Discussion: "Like in previous studies, we found that the prevalence of FH and PH of cancer varies by age, race/ethnicity, income and education, and sex." – can you cite which studies found these main observations?

Response: Thank you for highlighting the need to add citations, the citations for the two studies have now been added to this sentence.

12. This sentence is also missing a citation: "In CRC, previous studies have found that a higher proportion of patients with CRC diagnosed at 50 years of age or younger have a family history of the disease when compared to individuals diagnosed after this age."

Response: Thank you for asking for citations, two additional citations have been added to support this statement.

13. What were the limitations of your study? Was it the selection biases of AoU or NHIS recruitment that perhaps makes these two populations not comparable? Please state clearly in the discussion the limitations and biases inherent in your study.

Response: Thank you for the question. The main limitation in comparing the two populations (*AoU* and NHIS) is the method of recruitment. We highlighted in the 3rd and 4th paragraphs of the discussion that the AoU study required additional commitments (biosample submission, sharing of personal health records, etc.), recruitment occurred in participating clinics, and in general, the overall cohort is designed to include a higher proportion of underrepresented minorities while the family and personal history of cancer data is collected via surveys that are not required.

We believe the paper has been strengthened by the review process and the revisions made, and we hope you will now find it acceptable for publication in *PLOS ONE.* Please contact me at lbruce@ucsd.edu or (703) 409-1344 if I can answer any questions. Thank you very much for considering our manuscript.

Sincerely,

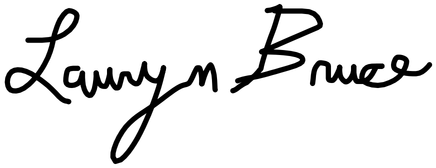

Lauryn Keeler Bruce, MS
